# Child marriage, maternal serum metal exposure, and risk of preterm birth in rural Bangladesh: evidence from mediation analysis

**DOI:** 10.1038/s41370-021-00319-3

**Published:** 2021-04-06

**Authors:** Hui Huang, Yongyue Wei, Yankai Xia, Liangmin Wei, Xin Chen, Ruyang Zhang, Li Su, Mohammad L. Rahman, Mahmudur Rahman, Quazi Qamruzzaman, Wenhui Guo, Hongbing Shen, Zhibin Hu, David C. Christiani, Feng Chen

**Affiliations:** 1 State Key Laboratory of Reproductive Medicine, Nanjing Medical University, Nanjing, China; 2 Department of Biostatistics, School of Public Health, Nanjing Medical University, Nanjing, China; 3 China International Cooperation Center for Environment and Human Health, Center of Global Health, Nanjing Medical University, Nanjing, China; 4 Key Laboratory of Modern Toxicology of Ministry of Education, School of Public Health, Nanjing Medical University, Nanjing, China; 5 Department of Environmental Health, Harvard T.H. Chan School of Public Health, Boston, MA USA; 6 Department of Population Medicine and Harvard Pilgrim Health Care Institute, Harvard Medical School, Boston, MA USA; 7 Dhaka Community Hospital Trust, Dhaka, Bangladesh; 8 Department of Epidemiology, School of Public Health, Nanjing Medical University, Nanjing, China

**Keywords:** Metal exposure, Child marriage, Preterm birth, Inductively coupled plasma mass spectrometry

## Abstract

**Background:**

The prevalence of preterm birth in Bangladesh is estimated to be 19.1%, the highest in the world. Although prenatal exposure to several metals has been linked with preterm birth, fewer prospective studies have investigated the socioeconomic factors that affect metal exposure, leading to preterm birth risk.

**Objective:**

We aim to identify novel metal biomarkers and their critical exposure windows, as well as the upstream socioeconomic risk factors for preterm birth in rural Bangladeshi, to shed light for future interventional strategies.

**Methods:**

This study included data from 780 mother–offspring pairs, who were recruited to participate in a prospective birth cohort in Bangladesh (2008–2011). Serum concentrations of 19 metals were measured in the first and second trimesters using inductively coupled plasma mass spectrometry. Mediation analysis was performed to explore the upstream socioeconomic factors that affect the risk of preterm birth mediated via metal exposure concentrations.

**Results:**

Early pregnancy exposure to serum zinc, arsenic, and strontium and mid-pregnancy exposure to barium were significantly associated with risk of preterm birth. Furthermore, younger marriage age was associated with an exponential increase in the risk of preterm birth, and women who married after 18 years old had a considerably lower risk of preterm birth. Mediation analysis indicated that these four elements mediated 30.2% of the effect of marriage age on preterm birth.

**Conclusion:**

This study indicated that maternal serum metal exposure mediates the impact of child marriage on the increased risk of preterm birth via metal exposures. The findings shed light on the mechanisms underlying such association and provide insights into future interventional strategies.

## Introduction

Preterm birth is associated with a high degree of immature organs. Infants born preterm (before 37 completed weeks of gestation [1]) are at greater risk of developing a range of short- and long-term comorbidities, especially lung dysplasia and infection [2]. Furthermore, preterm birth and its complications account for 35% of all neonatal mortality and they further account for ~16% of deaths in children under 5 years old [3]. About 15 million premature babies are born each year worldwide, 81.1% of whom are born in Asia and Sub-Saharan Africa [4]. In Bangladesh, the estimated national preterm birth prevalence is the highest in the world at 19.1% [4]. The rate is even higher in rural areas [5], posing a significant economic and emotional burden to families and the country.

Preterm birth results from the joint effects of various socioeconomic and demographic factors including, but not limited to, race, low socioeconomic status, multiple pregnancy, higher maternal age, history of abortion, pregnancy complications, and child marriage (marriage or union before the age of 18) [6]. At a certain level, pregnant women with a history of child marriage have fewer essential nutrients than pregnant women who are married normally [7]. Due to the living environment, working environment and income status, they have more opportunities to be exposed to harmful metal elements in the environment. Additionally, exposure to environmental toxicants is also linked to preterm birth. Environmental and industrial pollution and various anthropogenic activities in Bangladesh result in high exposures to several metals through multiple routes [8]. These exposures occur at levels that far exceed those in developed countries in Europe and North America as well as the safety threshold of the World Health Organization [9]. The effects of these exposures on birth outcomes have been investigated within a well-established birth cohort in Bangladesh [10]. Indeed, heavy metals and toxic elements including arsenic (As) [11], lead (Pb) [12], chromium (Cd) [13], and copper (Cu) [14] are associated with preterm birth and other adverse birth outcomes evaluated through hypothesis-driven studies [15–17]. In addition, previous studies have assessed the metals individually in relation to birth outcomes and at one specific timepoint during pregnancy [18]. However, few studies have explored the causal mechanism of the socioeconomic factors and the environmental and industrial pollution in human body on preterm birth.

Therefore, taking advantage of the well-established birth cohort in rural Bangladesh, we measured the concentrations of 19 metals in maternal serum samples simultaneously in the first and second trimesters using inductively coupled plasma mass spectrometry (ICP-MS). We aimed to identify novel metal markers, critical exposure windows, and used mediation analysis to identify upstream socioeconomic effects factors that affect metal exposure levels, thus increasing the risk of preterm birth in rural Bangladesh which will provide insights into future interventional strategies.

## Methods

### Study population

The Bangladesh birth cohort was established during 2008–2011, as previously described [10]. The inclusion criteria were: maternal age at current pregnancy ≥ 18 years, ultrasound-confirmed singleton pregnancy of ≤16 weeks gestation, used a tube well that supplied groundwater as their primary drinking water source, and planned to live at their current residence during the pregnancy. This study included 780 pregnant women with available serum samples in the first trimester. Of these, 610 participants had serum samples available in the second trimester. Informed consent was obtained from all participants before enrollment and prior to engaging in any use of data. Participants were also informed of the intention of this study, which was to determine how heavy metal exposure affects the health of pregnant women and their infants. Prenatal care and multivitamins were provided to all participants through community outreach clinics, which were among the few healthcare providers in the catchment areas. All protocols were reviewed and approved by the Human Research Committees at Harvard T.H. Chan School of Public Health (Boston, MA, USA), Nanjing Medical University (Nanjing, China), and Dhaka Community Hospital Trust (Dhaka, Bangladesh).

### Exposure assessment

Peripheral venous blood specimens were collected in EDTA tubes after an overnight fast. Samples were stored at 4 °C, shipped to the Trace Metals Laboratory at the Harvard Chan School, and subsequently centrifuged at 1600 × *g* for 10 min to obtain blood serum. All containers used in the experiments were cleaned by soaking in 10% nitric acid (HNO_3_) for 24 h and rinsing several times with 18 Ω deionized water. Before analysis, 60 μL serum was diluted to 1.8 mL with 1% (v/v) HNO_3_, 0.1% (v/v) Triton X-100, and 10 μg/L internal standards.

Nineteen metals were analyzed in the serum using the iCAP Qc ICP-MS system (Thermo Scientific, Bremen, Germany) at School of Public Health, Nanjing Medical University. The target metals included sodium (Na), magnesium (Mg), potassium (K), calcium (Ca), manganese (Mn), iron (Fe), cobalt (Co), copper (Cu), zinc (Zn), arsenic (As), rubidium (Rb), strontium (Sr), molybdenum (Mo), cadmium (Cd), antimony (Sb), barium (Ba), mercury (Hg), thallium (TI), and uranium (U). The protocol for metal analysis was adapted from a previous report [19]. The limit of detection (LOD) was calculated as three times the average of ten consecutive measurements of the blank diluent (0.1% [v/v] Triton X-100, 1% [v/v] HNO_3_ plus 10 μg/L internal standards including Sc, Y, In, Tb, and Bi; Appendix Table 1). Quality control samples were from Seronorm Trace Elements Serum L-2 (ref. 203113; Sero, Billingstad, Norway), and were analyzed in parallel with the study samples (every 20 study samples with one standard sample). The quality meets the normal quality control criteria. Metal concentrations below the LOD were imputed by LOD/2. All concentrations were log_e_ transformed before statistical analysis. Serum levels of Mn, Cd, and U were less than the LOD in more than 50% of the testing samples: Mn (63.5% for the first trimester, 52.0% for the second trimester), Cd (83.1% for the first trimester, 81.2% for the second trimester), and U (62.2% for the first trimester, 55.3% for the second trimester), which were additionally dichotomized to “detected” versus “non-detected” for subsequent analyses (Appendix Table 1).

### Outcome and covariates

Preterm birth is defined as a live birth before 37 completed weeks of gestation. Healthcare workers attended all births and collected detailed birth records. Gestational age was determined through ultrasonography by a licensed general practitioner using either the gestational sac mean diameter if the pregnancy was between 4 and 7 weeks, or the crown-rump length if the pregnancy was between 7 and 16 weeks [10]. Other demographic information including maternal age, maternal body mass index (BMI), marriage age, maternal and spouse education level, number of past pregnancies, family income level, and secondhand smoking exposure were collected at the time of enrollment using a structured questionnaire.

### Statistical analysis

Baseline characteristics of preterm cases and controls were compared using the *t*-test or Mann–Whitney *U* test for continuous variables depending on the data distribution (Appendix Figs. 1 and 2), and the chi-square test was used for categorical variables. Pearson correlation analysis (for normal distribution) and spearman rank correlation analysis (for skewed distribution) were used to evaluate correlations among the log_e_ transformed serum metal concentrations in the first (Appendix Fig. 3) and second trimesters (Appendix Fig. 4) and the correlation matrix of serum metal exposure between the first and second trimesters (Appendix Fig. 5).

To identify the potential effects of metal exposure on risk of preterm birth, mixed-effects logistic models with repeated measures for log_e_ transformed metal concentrations from both trimesters were performed to estimate the odds ratio (OR), 95% confidence interval (CI), and *P* value for the risk of preterm birth. Sociodemographic factors, which were differentially distributed in the preterm birth and normal birth groups (Table 1), were considered covariates of the statistical models including maternal age, marriage age, secondhand smoking, BMI categories, parents’ education levels, and family income level. False discovery rate (FDR) was used to control for multiple comparisons, and FDR-*q* value < 0.05 was considered statistically significant. To investigate the sensitive exposure windows for candidate metals, logistic regression models were separately performed for each metal in each trimester, to estimate the OR, 95% CI, and *P* values for individual serum metals adjusting for the same set of covariates.

**Table 1:** Demographic, social-economic, and birth characteristics of the study participants.

Variable	Overall (*n* = 780)	Term birth (*n* = 605)	Preterm birth (*n* = 175)	*P*
Baseline age (years)	22.72 ± 4.01	22.69 ± 4.00	22.83 ± 4.06	0.7^a^
BMI (kg/m^2^)	20.42 ± 3.16	20.49 ± 3.27	20.19 ± 2.74	0.2^a^
Weight (kg)	55.2 ± 8.05	55.99 ± 8.14	52.46 ± 7.09	4.4 × 10^−08a^
Height (cm)	150.97 ± 5.39	151.26 ± 5.31	149.98 ± 5.58	7.5 × 10^−03a^
Marriage age (years)	17.46 ± 2.31	17.76 ± 2.25	16.43 ± 2.19	1.4 × 10^−11a^
Child marriage				
No	434 (55.6%)	383 (63.3%)	51 (29.1%)	2.3 × 10^−15c^
Yes	346 (44.4%)	222 (36.7%)	124 (70.9%)	
Education				
No formal education	106 (13.6%)	76 (12.6%)	30 (17.1%)	1.2 × 10^−2b^
Primary education	252 (32.3%)	211 (34.9%)	41 (23.4%)	
Secondary or higher	422 (54.1%)	318 (52.6%)	104 (59.4%)	
Spouse education				
No formal education	201 (25.8%)	139 (23.0%)	62 (35.4%)	4.4 × 10^−05b^
Primary education	251 (32.2%)	217 (35.9%)	34 (19.4%)	
Secondary or higher	328 (42.1%)	249 (41.2%)	79 (45.1%)	
Secondhand smoking				
No	465 (59.6%)	369 (61.0%)	96 (54.9%)	0.2^c^
Yes	315 (40.4%)	236 (39.0%)	79 (45.1%)	
Income				
≤4000 Taka	354 (45.4%)	251 (41.5%)	103 (58.9%)	1.2 × 10^−04b^
4001–6000 Taka	334 (42.8%)	273 (45.1%)	61 (34.9%)	
≥6000 Taka	86 (11.0%)	76 (12.6%)	10 (5.7%)	
Number of previous pregnancies				
0	310 (39.7%)	264 (43.6%)	64 (36.6%)	0.2^b^
1	239 (30.1%)	183 (30.2%)	56 (32.0%)	
≥6	213 (27.3%)	158 (26.1%)	55 (31.4%)	
Birth type				
Vaginal	494 (63.3%)	364 (60.2%)	130 (74.3%)	9.0 × 10^−4c^
Cesarean	286 (36.7%)	241 (39.8%)	45 (25.7%)	
Birth place				
Home	413 (52.9%)	305 (50.7%)	108 (61.7%)	3.7 × 10^−4b^
Clinic	54 (6.9%)	38 (6.3%)	16 (9.1%)	
Hospital	310 (39.7%)	259 (43.0%)	51 (29.1%)	
Child sex				
Male	398 (51.0%)	314 (51.9%)	84 (48.0%)	0.4^c^
Female	382 (49.0%)	291 (48.1%)	91 (52.0%)	
Gestational age (weeks)	37.9 ± 2.06	38.76 ± 1.15	34.94 ± 1.70	9.2 × 10^−75a^

Continuous variables are presented as mean ± standard deviation; categorical variables are presented as frequency and proportion [*n* (%)].

^a^*P* value was derived from the Student’s *t* test.

^b^*P* value was derived from the rank-sum test.

^c^*P* value was derived from the *χ*^2^ test.

In addition, partial correlation analysis was performed to evaluate the effects of baseline socioeconomic factors on individual metal concentrations for the first trimester and second trimesters. Marriage age was significantly associated with the concentrations of all candidate metals. Mediation analysis was performed to determine whether marriage age affects the risk of preterm birth via metal exposure concentrations [20]. The paradigm of mediation analysis in Appendix Fig. 6 explains the conceptual model for causal mediation analysis [21], which comprised two separate models: model for preterm birth (outcome model, Model 1) and model for metal exposure (mediator model, Model 2)

1 $${\mathrm{Logit}}\left( {{\uppi }}_{i} \right) = {\uptheta}_0 + {\uptheta}_1\left( {{\mathrm{Marriage}}\,{\mathrm{age}}} \right)_{i} \,+ \,{\uptheta}_2\ln \left( {{\mathrm{metal}}} \right)_{i} \,+ \,{\uptheta}_3{C}_{i}$$

2 $${E}\left( {\ln \left( {{\mathrm{metal}}} \right)_{i}} \right) = {\upbeta}_0 + {\upbeta}_1\left( {{\mathrm{Marriage}}\,{\mathrm{age}}} \right)_{i} \,+\, {\upbeta}_2{C}_{i}$$

Notation *π*_*i*_ denotes the probability of preterm birth, and *C*_*i*_ denotes the covariate matrix for subject *i*. The two models were combined to decompose the total effect of marriage age on the risk of preterm birth into two parts: indirect effect, which represents the effect of marriage age mediated via metal exposure, and direct effect, which represents the effect of marriage age mediated via pathways independent of metal exposure. The total effect, estimated as the sum of the direct and indirect effects, represented the overall effect of marriage age on preterm birth. Analyses were conducted using R software Version, v3.6.2 (The R Foundation for Statistical Computing), and two-sided *P* < 0.05 was considered statistically significant unless stated otherwise.

## Results

### Characteristics of the study population and elements

Demographics of the 780 mothers are described in Table 1. Mean marriage age was 17.46 ± 2.31 years and maternal age was 22.72 ± 4.01 years old. The proportion of participants married before 18 years old was 44.4% in the study population. Of 780 singleton livebirths, 175 (22.4%) were born preterm. Compared with term births, women giving birth preterm were more likely to have gotten married before 18 years old and have secondhand smoking exposure, with lower maternal baseline weight, parent’s educational level, and household income (Table 1). Among the 19 elements, with the exception of Ca, Na, Mg, and K that were highly correlated with each other, most were modestly correlated in the first and second trimesters, and across trimesters (Appendix Figs. 3–5). In addition, concentrations of 16/19 metals significantly changed between the two trimesters (Appendix Table 1).

### Critical metals and exposure window for the risk of preterm birth

**Fig. 1:**
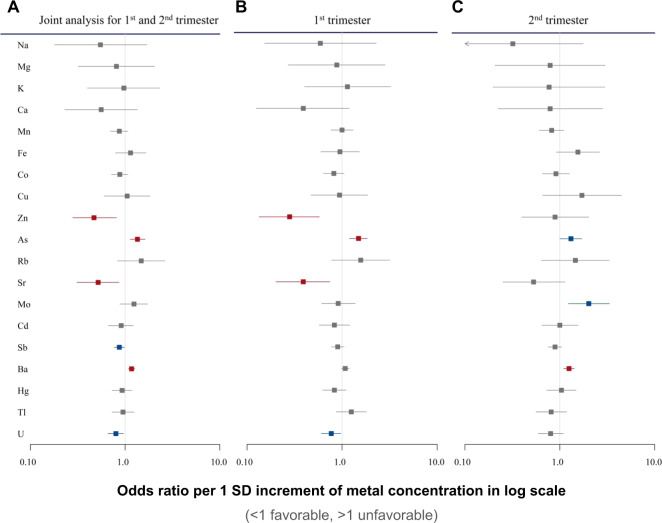
Maternal serum metal concentrations and risk of preterm birth. **A** Forest plots of OR and 95% CI for the relationship of serum metal concentrations in the first and second trimesters with preterm birth were estimated using the mixed effect model. Model was adjusted for baseline age, BMI, secondhand smoking status, education, spouse education, income levels, marriage age, and number of previous pregnancies. **B** Forest plots of OR and 95% CI for the relationship of serum metal concentrations in the first trimester with preterm birth were estimated using the logistic model. Model was adjusted for baseline age, BMI, secondhand smoking status, education, spouse education, income levels, marriage age, and number of previous pregnancies. **C** Forest plots of OR and 95% CI for the relationship of serum metal concentrations in the second trimester with preterm birth were estimated using the logistic model. Model was adjusted for baseline age, BMI, secondhand smoking status, education, spouse education, income levels, marriage age, and number of previous pregnancies.

Using mixed-effects logistic models incorporating metal concentrations of two timepoints as repeated measures, four metals (Zn, As, Sr, and Ba) were significantly associated with the risk of preterm birth (FDR-*q* value < 0.05) (Fig. 1A and Appendix Table 2). We analyzed concentrations of these 19 metals in the first and second trimesters to determine critical exposure timing. Concentrations of serum Zn (OR = 0.28; 95% CI: 0.13–0.58; *P* = 6.0 × 10^−04^, FDR-*q* = 5.7 × 10^−03^), As (OR = 1.49; 95% CI: 1.20–1.84; *P* = 3.0 × 10^−04^, FDR-*q* = 5.7 × 10^−03^), and Sr (OR = 0.39; 95% CI: 0.20–0.74; *P* = 4.3 × 10^−03^, FDR-*q* = 2.7 × 10^−02^) in the first trimester were significantly associated with preterm birth (Fig. 1B and Appendix Table 2). On the other hand, only Ba retained significant association with the risk of preterm birth (OR = 1.25; 95% CI: 1.10–1.41; *P* = 7.0 × 10^−04^) (Fig. 1C and Appendix Table 2) in the second trimester. These results show that maternal exposure to Zn, As, Sr, and Ba and their crucial exposure windows increase the risk of preterm birth.

### Marriage age, metal exposure, and preterm birth

**Fig. 2:**
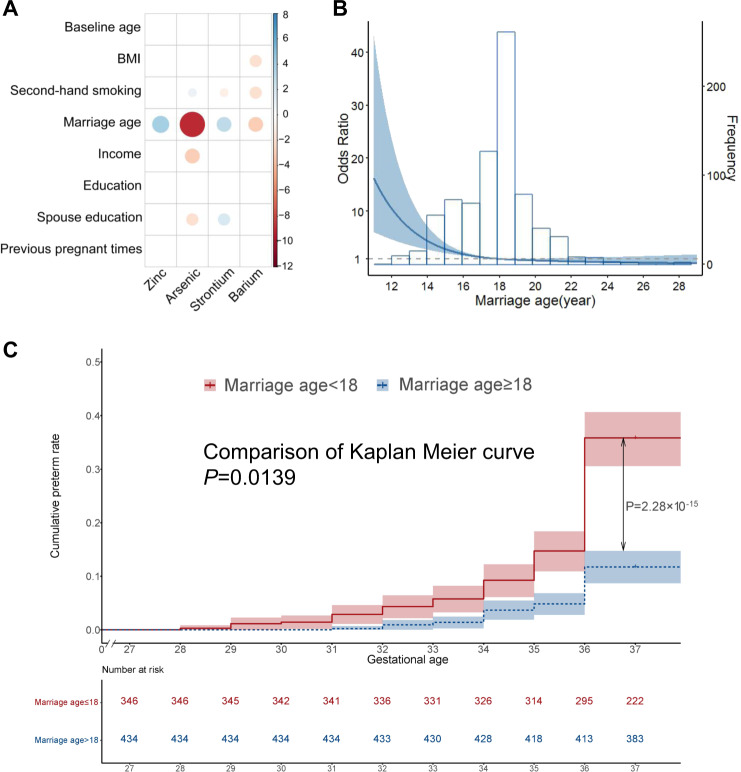
Relationship among social-economic factors, maternal metal exposure, and risk of preterm birth. **A** Correlation between social-economic factors and four significant metal elements. Blue dots indicate positive regression coefficients and red dots indicate negative regression coefficients, where the position of the color indicates that the correlation is significant and larger circles indicate greater significance (−log10 (*P* value)). **B** The restricted cubic spline for the relationship between marriage age and preterm birth. The lines represent adjusted ORs based on restricted cubic splines for the marriage age in the conditional logistic model. Adjustment factors were baseline age, BMI, secondhand smoking status, education, income levels, and number of previous pregnancies. **C** Kaplan–Meier survival curves of cumulative preterm birth rate according to marriage age > 18 years old and ≤18 years old.

**Fig. 3:**
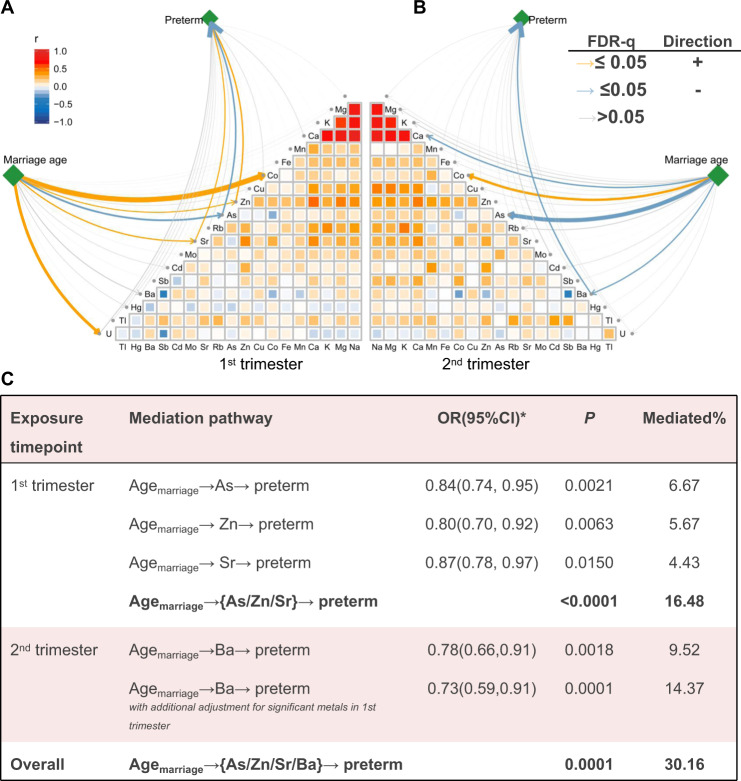
Relationship among marriage age, metal exposure, and risk of preterm birth. Relationship among marriage age, metal exposure, and risk of preterm birth in the first (**A**) and second trimesters (**B**). The yellow line indicates that the FDR is <0.05 and is positive, the blue line indicates that the FDR is <0.05 and is negative, and the gray line indicates that the FDR is larger than 0.05. **C** OR and 95% CI represent the indirect effects of marriage age on risk of preterm mediated through metal elements; the effects of marriage age on preterm birth were mediated by maternal serum.

Child marriage of girls (marriage age < 18 years old) is a risk factor for preterm birth [6]. In this study, restricted cubic spline analysis for continuous marriage age showed a more detailed exponential dose-response relationship with the risk of preterm birth (Fig. 2B). The optimal cutoff for marriage age was estimated to be ~18 years old. Notably, the exponential dose-response relationship mainly existed in women married before 18 years old. Women who married after 18 years old had a considerably lower risk of preterm birth, which was adjuster for BMI categories, parents’ education levels, and family income level for potential confounding effects (Fig. 2C). Notably, marriage age showed an independent and considerable significant correlation with all four metals, which was associated with preterm birth after adjusting for the other sociodemographic factors (Fig. 2A). We have performed partial correlation analysis to evaluate the effects of marriage age on individual metal concentrations for the first trimester and second trimesters (Appendix Table 3) and found that marriage age was significantly associated with the concentrations of candidate metals (Zn: *β* = 0.01 (0.00, 0.02), *P* = 3.9 × 10^−03^; As: *β* = −0.06(−0.09, −0.03), *P* = 1.4 × 10^−04^; Sr: *β* = 0.02 (0.01, 0.03), *P* = 1.6 × 10^−03^ for the first trimester; Ba: *β* = −0.11 (−0.18, −0.03), *P* = 0.01 for the second trimester) (Appendix Table 3). To determine whether maternal metal concentrations mediate the effect of marriage age on preterm birth, we conducted causal mediation analysis to analyze the potential causal relationships among child marriage (exposure), metal exposure (mediators), and risk of preterm birth (outcome) (Fig. 3A, B). Mediation analysis showed that the causal effect of marriage age on preterm birth was mediated by maternal serum Zn (OR = 0.84 per 5-year increase in marriage age; 95% CI: 0.74–0.95; *P* = 2.1 × 10^−03^; proportion mediated, 6.67%), As (OR = 0.80; 95% CI: 0.70–0.92; *P* = 6.3 × 10^−03^; proportion mediated, 5.67%), and Sr (OR = 0.87; 95% CI: 0.78–0.97; *P* = 1.5 × 10^−02^; proportion mediated, 4.43%) during the first trimester (Fig. 3C). These three metals together mediated 16.48% of the effect of marriage age. On the other hand, Ba in the second trimester was also identified as a mediator (OR = 0.78; 95% CI: 0.66–0.91; *P* = 1.8 × 10^−03^; proportion mediated, 9.52%) (Appendix Table 4). These four elements together mediated 30.16% of the effect of marriage age on preterm birth (Fig. 3C), indicating that marriage age has a considerable impact on preterm birth by affecting maternal metal exposure levels.

## Discussion

In this study, dozens of maternal serum metals were repeatedly evaluated at two timepoints during pregnancy in a prospective birth cohort in Bangladesh. Serums Zn and Sr in the first trimester appeared to be protective against preterm birth, whereas serum As in the first trimester was positively associated with the increased risk of preterm birth. In addition, serum Ba in the second trimester was associated with the increased risk of preterm birth. Furthermore, marriage age was associated with an exponential increase in the risk of preterm birth, and women married after age 18 years old had a considerably lower risk of preterm birth. Mediation analysis indicated that these four elements together mediated nearly one-third (~30%) of the effect of marriage age on preterm birth.

Not surprisingly, As concentration in maternal serum was significantly associated with the risk of preterm birth, which was more evident in women with a history of child marriage. As has been well studied for its impact on adverse birth outcomes, including preterm birth [22]. As exposure from drinking water has been the dominant route of exposure in the past decades [23]. Remediation activities and safe water programs including testing tube wells, labeling unsafe wells, and installing new As-free water sources and point-of-use filters have successfully reduced As exposure in the population [24]. The result is of great significance for maternal-child health and public health to optimize fertility [25]. However, the proportion of people exposed to As remains high and has slowly decreased just in recent years [26].

Our study quantitatively analyzed and thoroughly illustrated the contribution of marriage age on the risk of preterm birth by a nonlinear statistical method, and identified 18 years old as the optimal minimum marriage age with respect to risk of preterm birth. Child marriage impacts metal exposure during pregnancy and mediates the hazard by altering the concentrations of these metals. Child marriage has been illegal in Bangladesh since adoption of the Child Marriage Restraint Act of 1929. However, the law is poorly enforced because of mild punishment. Furthermore, the latest Bangladesh Child Marriage Restraint Act 2017 allows marriage under 18 in “special cases,” which would be a step backwards for Bangladesh and may result in an even higher rate of child marriage. More than half of girls from the poorest families in the developing world are married as children. Several qualitative studies have reported that adolescent mothers have a higher likelihood of spontaneous abortion, preterm birth, fetal death, and infant death [27–29]. Our study further confirmed the necessity of banning child marriage. Importantly, the metals together mediate ~30% of the risk of marriage age on the outcome. At a certain level, pregnant women with a history of child marriage have fewer essential nutrients than pregnant women who are married normally [7]. Previous studies have shown that pregnant women are very prone to Zn deficiency, especially in undeveloped countries, such as Bangladesh [30]. Pregnant women with a history of child marriage may be more susceptible to the intrusion of toxic heavy metals in the harsh environment, leading to adverse pregnancy outcomes and unable to obtain adequate pregnancy nutrition supplement. While the prevalence of child marriage has decreased worldwide, the harmful practice remains widespread due to economic status or regional culture, especially in underdeveloped countries.

To assess the proportion of preterm birth that could be potentially prevented if risk factors were removed, the population attributable risk percentage (PAR%) was calculated for each important risk factor. Child marriage had 3.18 times higher risk (95% CI: 1.78, 5.29) of preterm birth, and the estimated PAR% was 54.23% (95% CI: 42.91, 65.55%). Therefore, it can be seen that reducing child marriage in the society can improve most of the preterm births. In addition, candidate metals found in our study also showed significant PAR%, including As: PAR% = 21.02% (2.14, 39.90%), Zn: −30.24% (−59.35, −1.14%), and Sr: −19.19% (−45.11, −6.73%) for the first trimester and Ba: PAR% = 27.31% (7.95, 46.68%) for the second trimester (Appendix Table 5). Thus, reducing the exposure of heavy metals in the environment and reducing child marriage are both important protective measures to improve preterm birth. The two are complementary, because the two-pronged approach to jointly formulate corresponding public health strategies, such as testing tube wells, labeling unsafe wells, and installing new arsenic-free water sources and point-of-use filters, is important for Bangladesh. The improvement of preterm birth in the region has important social value.

This study had some limitations. First, because each metal has a unique distribution in the organs and circulatory system, this study only tested metal concentrations in the serum, which may not be a suitable biomarker for the internal exposure of all metals [31]. For example, serum metals are useful and effective for assessing the exposure status of certain metals (e.g., serum As), while urine or whole blood concentrations are commonly used for other metals (e.g., urinary As and whole blood Pb). Second, ~21.8% of participants in our cohort did not have serum samples in the second trimester, which reduced statistical power. However, the likelihood of bias is small in terms of baseline demographic characteristics. Last, the study is lack of a systematic assessment of nutritional status during pregnancy, which may affect exposure and outcome. Therefore, we could not estimate the contribution and interaction of nutritional status during pregnancy to the relationship between prenatal exposure to metals and gestational age.

## Conclusion

This study identified four maternal serum metal biomarkers that are jointly associated with the risk of preterm birth at crucial exposure windows during pregnancy, and jointly mediate about one-third of the risk effect of child marriage. This finding suggests that child marriage may be a modifiable factor that affects premature birth, providing support for promoting later marriage age to protect maternal-child health.

## Supplementary information

Supplement Table R1

## References

[1] Mwaniki MK, Atieno M, Lawn JE, Newton CR (2012). Long-term neurodevelopmental outcomes after intrauterine and neonatal insults: a systematic review. Lancet.

[2] Chawanpaiboon S, Vogel JP, Moller AB, Lumbiganon P, Petzold M, Hogan D (2019). Global, regional, and national estimates of levels of preterm birth in 2014: a systematic review and modelling analysis. Lancet Glob Health.

[3] Liu L, Oza S, Hogan D, Perin J, Rudan I, Lawn JE (2015). Global, regional, and national causes of child mortality in 2000-13, with projections to inform post-2015 priorities: an updated systematic analysis. Lancet.

[4] Blencowe H, Cousens S, Oestergaard MZ, Chou D, Moller AB, Narwal R (2012). National, regional, and worldwide estimates of preterm birth rates in the year 2010 with time trends since 1990 for selected countries: a systematic analysis and implications. Lancet.

[5] Shah R, Mullany LC, Darmstadt GL, Mannan I, Rahman SM, Talukder RR (2014). Incidence and risk factors of preterm birth in a rural Bangladeshi cohort. BMC Pediatr.

[6] Pandya YP, Bhanderi DJ (2015). An epidemiological study of child marriages in a rural community of Gujarat. Indian J Community Med.

[7] Efevbera Y, Bhabha J, Farmer PE, Fink G (2017). Girl child marriage as a risk factor for early childhood development and stunting. Soc Sci Med.

[8] Meharg AA, Norton G, Deacon C, Williams P, Adomako EE, Price A (2013). Variation in rice cadmium related to human exposure. Environ Sci Technol.

[9] Evaluation of certain food additives and contaminants. Twenty-second report of the joint FAO/WHO Expert Committee on Food Additives. World Health Organ Tech Rep Ser. 1978:1–39. 107662

[10] Rahman ML, Kile ML, Rodrigues EG, Valeri L, Raj A, Mazumdar M (2018). Prenatal arsenic exposure, child marriage, and pregnancy weight gain: associations with preterm birth in Bangladesh. Environ Int.

[11] Kile ML, Houseman EA, Breton CV, Smith T, Quamruzzaman Q, Rahman M (2007). Dietary arsenic exposure in Bangladesh. Environ Health Perspect.

[12] Tsuji M, Shibata E, Morokuma S, Tanaka R, Senju A, Araki S (2018). The association between whole blood concentrations of heavy metals in pregnant women and premature births: the Japan Environment and Children’s Study (JECS). Environ Res.

[13] Freire C, Amaya E, Gil F, Murcia M, S LL, Casas M (2019). Placental metal concentrations and birth outcomes: the Environment and Childhood (INMA) project. Int J Hyg Environ Health.

[14] Irwinda R, Wibowo N, Putri AS (2019). The concentration of micronutrients and heavy metals in maternal serum, placenta, and cord blood: a cross-sectional study in preterm birth. J Pregnancy.

[15] Welch BM, Branscum A, Ahmed SM, Hystad P, Smit E, Afroz S (2019). Arsenic exposure and serum antibody concentrations to diphtheria and tetanus toxoid in children at age 5: A prospective birth cohort in Bangladesh. Environ Int.

[16] Obrycki JF, Lee JJ, Kapur K, Paul L, Hasan M, Mia S, et al. A case-control analysis of maternal diet and risk of neural tube defects in Bangladesh. Birth Defects Res. 2019. 10.1002/bdr2.1505. 10.1002/bdr2.1505 PMC6703921 30989821

[17] Ahmed SM, Noble BN, Joya SA, Ibn Hasan MOS, Lin PI, Rahman ML (2019). A PRospective Cohort Study Examining the Associations of Maternal Arsenic Exposure with Fetal Loss and Neonatal Mortality. Am J Epidemiol.

[18] Hao Y, Pang Y, Yan H, Zhang Y, Liu J, Jin L (2019). Association of maternal serum copper during early pregnancy with the risk of spontaneous preterm birth: a nested case-control study in China. Environ Int.

[19] Silver MK, Arain AL, Shao J, Chen M, Xia Y, Lozoff B (2018). Distribution and predictors of 20 toxic and essential metals in the umbilical cord blood of Chinese newborns. Chemosphere.

[20] Imai K, Keele L, Tingley D (2010). A general approach to causal mediation analysis. Psychol Methods.

[21] Hernan MA (2004). A definition of causal effect for epidemiological research. J Epidemiol Community Health.

[22] Bloom MS, Surdu S, Neamtiu IA, Gurzau ES (2014). Maternal arsenic exposure and birth outcomes: a comprehensive review of the epidemiologic literature focused on drinking water. Int J Hyg Environ Health.

[23] Vahter M (2009). Effects of arsenic on maternal and fetal health. Annu Rev Nutr.

[24] Seow WJ, Pan WC, Kile ML, Baccarelli AA, Quamruzzaman Q, Rahman M (2012). Arsenic reduction in drinking water and improvement in skin lesions: a follow-up study in Bangladesh. Environ Health Perspect.

[25] Hall M, Gamble M, Slavkovich V, Liu X, Levy D, Cheng Z (2007). Determinants of arsenic metabolism: blood arsenic metabolites, plasma folate, cobalamin, and homocysteine concentrations in maternal-newborn pairs. Environ Health Perspect.

[26] Jamil NB, Feng H, Ahmed KM, Choudhury I, Barnwal P, van Geen A (2019). Effectiveness of different approaches to arsenic mitigation over 18 years in Araihazar, Bangladesh: implications for national policy. Environ Sci Technol.

[27] Chen XK, Wen SW, Fleming N, Demissie K, Rhoads GG, Walker M (2007). Teenage pregnancy and adverse birth outcomes: a large population based retrospective cohort study. Int J Epidemiol.

[28] Fraser AM, Brockert JE, Ward RH (1995). Association of young maternal age with adverse reproductive outcomes. N Engl J Med.

[29] Shawky S, Milaat W (2000). Early teenage marriage and subsequent pregnancy outcome. East Mediterr Health J.

[30] Wang H, Hu YF, Hao JH, Chen YH, Wang Y, Zhu P (2016). Maternal serum zinc concentration during pregnancy is inversely associated with risk of preterm birth in a Chinese population. J Nutr.

[31] Smith D, Hernandez-Avila M, Tellez-Rojo MM, Mercado A, Hu H (2002). The relationship between lead in plasma and whole blood in women. Environ Health Perspect.

