# Supplementary material for: Child marriage, maternal serum metal exposure, and risk of preterm birth in rural Bangladesh: evidence from mediation analysis
Source: J Expo Sci Environ Epidemiol. 2021 Apr 6;31(3):571–80. doi: 10.1038/s41370-021-00319-3 (PMC8134042; doi:10.1038/s41370-021-00319-3)
Supplement: Supplementary file 1 — Supplement Table R1 [file 41370_2021_319_MOESM1_ESM.docx]

**Supplementary Appendix**

**Title:** **Child Marriage, Maternal Serum Metal Exposure, and Risk of Preterm Birth in Rural Bangladesh: Evidence from Mediation Analysis**

**Contents**

**Appendix Figures**

**[Appendix Figure 1. Distribution of metal concentrations in the first trimester.](#_Toc1271) 3**

**[Appendix Figure 2. Distribution of metal concentrations in the second trimester.](#_Toc11371) 5**

**[Appendix Figure 3. Correlation matrix of serum metal exposures in the first trimester.](#_Toc17173) 6**

**[Appendix Figure 4. Correlation matrix of serum metal exposures in the second trimester.](#_Toc3556) 7**

**[Appendix Figure 5. Correlation matrix of serum metal exposures between the first and second trimesters.](#_Toc9918) 8**

**[Appendix Figure 6. Generic paradigm for mediation analysis.](#_Toc3556) 13**

**Appendix Tables**

**[Appendix Table 1. Summarized results of metal exposure levels in the first and second trimesters.](#_Toc12263) 4**

**[Appendix Table 2. Relationship between maternal metal exposure in the first or second trimester and the risk of preterm birth (binary outcome).](#_Toc16205) 9**

**[Appendix Table 3. The effects of marriage age on individual metal concentrations for the first trimester and second trimesters.](#_Toc16205) 14**

**[Appendix Table 4. Indirect effects of marriage age on risk of preterm mediated through metal elements.](#_Toc16205) 17**

**[Appendix Table 5. Odds ratio, PAR% and NNT of child marriage and metal exposure on risk of preterm birth.](#_Toc16205) 18**

**Appendix Figure 1. Distribution of metal concentrations in maternal serum in the first trimester.**

**Appendix Table 1.** **Summarized results of metal exposure levels in the first and second trimesters.**

| **Metal** | **LOD^a^** | **LOQ^b^** | **First trimester** | | **Second trimester** | | Difference^c^ | *P*^c^ |
| --- | --- | --- | --- | --- | --- | --- | --- | --- |
|  |  |  | Median (Q_1_, Q_3_) | *N* (%) _<LOD_ | Median (Q_1_, Q_3_) | *N*(%) _of <LOD_ |  |  |
| Na | 0.5951 | 1.9838 | 3304.42 (3054.90,3638.26) | 0 | 3394.83 (3171.71,3641.11) | 1 (0.2%) | 47.4027 | 6.3×10^-02^ |
| Mg | 0.2981 | 0.9937 | 18.96 (17.17,21.06) | 0 | 18.69 (17.15,20.43) | 3 (0.5%) | -0.4573 | 3.5×10^-03^ |
| K | 0.2374 | 0.7912 | 138.79 (124.76,157.75) | 0 | 147.77 (135.14,163) | 0 | 6.2669 | 7.8×10^-06^ |
| Ca | 1.7625 | 5.8749 | 81.75 (73.83,90.16) | 0 | 83.21 (75.17,89.56) | 1 (0.2%) | -0.3010 | 0.7 |
| Mn | 1.4584 | 4.8615 | 0.73 (0.73,1.97) | 496 (63.5%) | 0.73 (0.73,2.17) | 345 (52.0%) | 0.2254 | 1.6×10^-02^ |
| Fe | 43.5105 | 145.0349 | 996.85 (775.96,1273.95) | 0 | 891.40 (680.17,1195.93) | 1 (0.2%) | -74.0730 | 2.3×10^-03^ |
| Co | 0.0731 | 0.2436 | 0.19 (0.11,0.29) | 81 (10.4%) | 0.30 (0.19,0.46) | 12 (1.8%) | 0.1440 | 6.0×10^-44^ |
| Cu | 5.9681 | 19.8936 | 1391.69 (1157.43,1666.05) | 0 | 1685.02 (1486.72,1946.10) | 3 (0.5%) | 294.5183 | 1.5×10^-43^ |
| Zn | 86.3765 | 287.9215 | 634.01 (537.23,757.50) | 0 | 525.85 (444.85,630.32) | 1 (0.2%) | -116.8535 | 1.4×10^-34^ |
| As | 0.3115 | 1.0385 | 0.99 (0.64,1.85) | 40 (5.1%) | 1.1 (0.70,2.16) | 21 (3.2%) | 0.1226 | 2.1×10^-02^ |
| Rb | 0.64564 | 2.1521 | 334.04 (272.73,399.95) | 0 | 351.02 (291.42,414.72) | 3 (0.5%) | 13.2105 | 1.8×10^-03^ |
| Sr | 4.5884 | 15.2946 | 39.20 (32.44,47.01) | 0 | 44.66 (36.70,53.83) | 2 (0.3%) | 5.6056 | 1.1×10^-21^ |
| Mo | 0.1842 | 0.6140 | 2.17 (1.60,2.96) | 0 | 2.57 (1.82,3.42) | 1 (0.2%) | 0.4113 | 1.1×10^-09^ |
| Cd | 0.0842 | 0.2806 | 0.04 (0.04,0.04) | 649 (83.1%) | 0.04 (0.04,0.04) | 539 (81.2%) | -0.0039 | 0.3 |
| Sb | 0.7528 | 2.5095 | 3.07 (0.38,5.02) | 195 (25.0%) | 4.12 (0.47,7.06) | 165 (24.9%) | 1.1751 | 1.2×10^-13^ |
| Ba | 2.6644 | 8.8812 | 67.54 (1.33,96.82) | 242 (31.0%) | 75.81 (1.33,102.83) | 165 (24.9%) | 8.6798 | 1.5×10^-03^ |
| Hg | 0.0929 | 0.3098 | 0.61 (0.44,0.89) | 1 (0.1%) | 0.60 (0.44,0.86) | 3 (0.5%) | -0.1360 | 1.2×10^-02^ |
| Tl | 0.0076 | 0.0252 | 0.02 (0.02,0.03) | 8 (1.0%) | 0.03 (0.02,0.04) | 2 (0.3%) | 0.0053 | 3.2×10^-10^ |
| U | 0.0168 | 0.0558 | 0.01 (0.01,0.03) | 486 (62.2%) | 0.01 (0.01,0.03) | 367 (55.3%) | -0.0095 | 2.6×10^-08^ |

^a^ LOD was defined as 3 times the average of 10 calibration blank solution samples.

^b^ LOQ was defined as 10 times the average of 10 calibration blank solution samples.

^c^ Average change between the first and second trimesters and the corresponding *P* value for the paired *t*-test.

**Appendix Figure 2. Distribution of metal concentrations in maternal serum in the second trimester.**

**Appendix Figure 3. Correlation matrix of serum metal exposure in the first trimester.**

The numbers in the cells of the lower triangle represent correlation coefficients (*r*) of the corresponding metals. Blue and red dots in the cells of the upper triangle indicate a positive and negative correlation, respectively; the diameter of the circle is proportional to the correlation coefficient. The cells devoid of numbers or dots indicate no significant correlation between the corresponding metals.

**
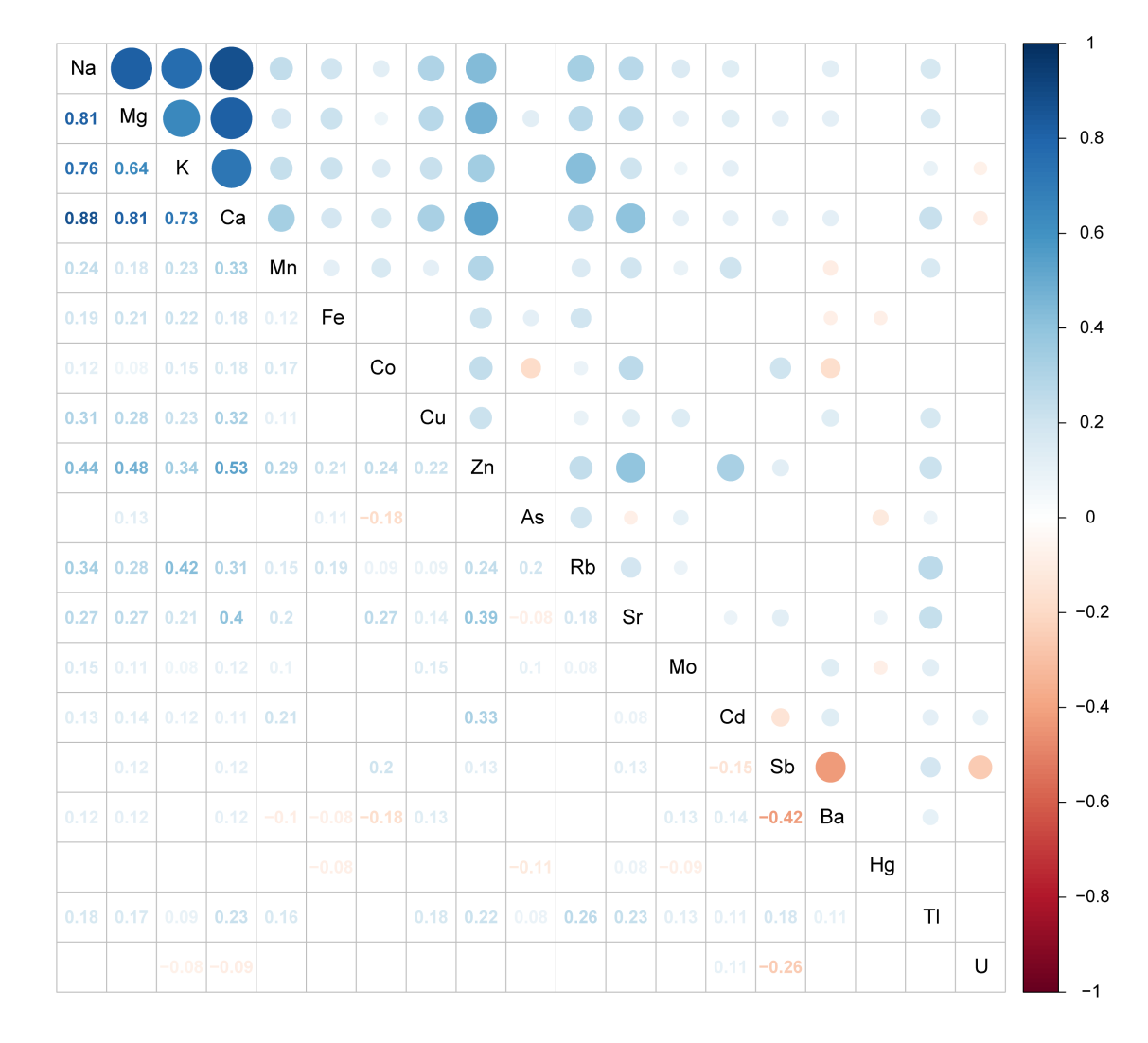
**

**Appendix Figure 4. Correlation matrix of serum metal exposure in the second trimester.**

The numbers in the cells of the lower triangle represent correlation coefficients (*r*) of the corresponding metals. Blue and red dots in the cells of the upper triangle indicate a positive and negative correlation, respectively; the diameter of the circle is proportional to the correlation coefficient. The cells devoid of numbers or dots indicate no significant correlation between the corresponding metals.

**
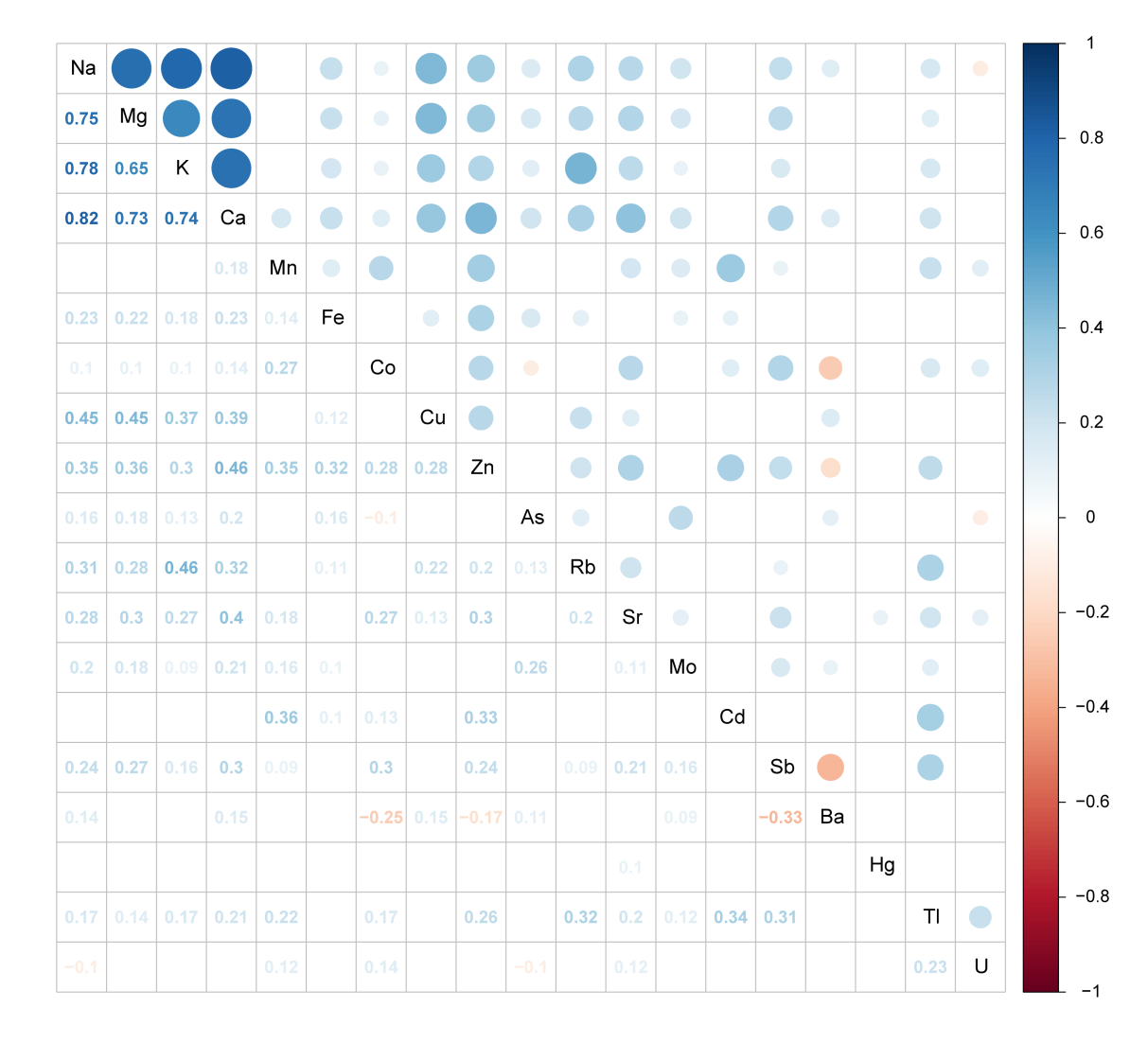
**

**Appendix Figure 5.** **Correlation matrix of serum metal exposure between the two trimesters.**

M1: first trimester, M2: second trimester. Blue and red dots in the cells of the upper triangle indicate a positive and negative correlation, respectively; the diameter of the circle is proportional to the correlation coefficient. The cells devoid of numbers or dots indicate no significant correlation between the corresponding metals.

**
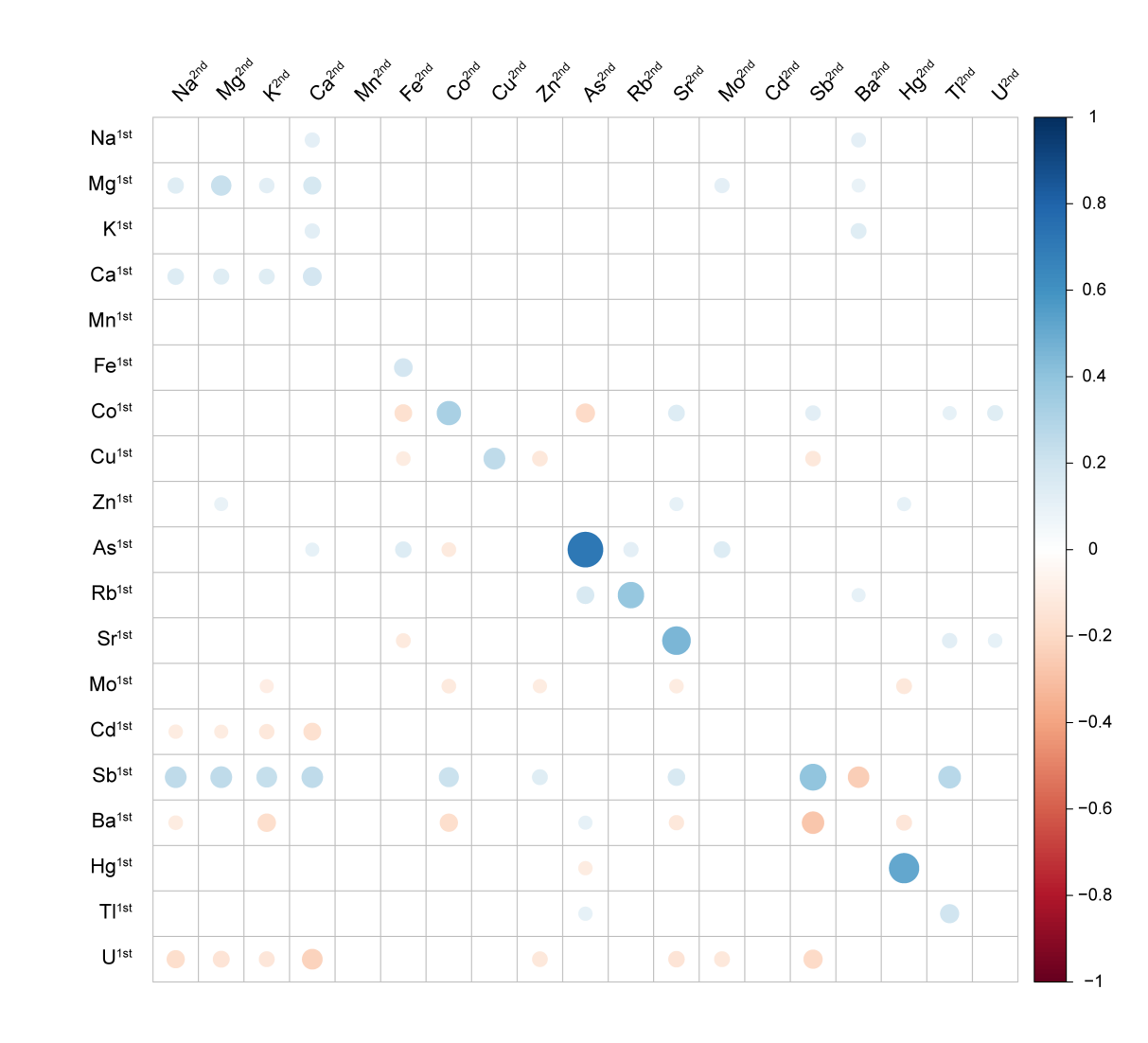
**

**Appendix Table 2.** **Relationship between maternal metal exposure in the first or second trimester and the risk of preterm birth.**

| **Metals** | **Joint analysis for**  **first and second trimester** | | **First trimester** | | **Second trimester** | |
| --- | --- | --- | --- | --- | --- | --- |
|  | OR (95%CI) | *P* | OR (95%CI) | *P* | OR (95%CI) | *P* |
| Na | 3373.64 (3118.26,3670.84) |  | 3304.42 (3054.90,3638.26) |  | 3394.83 (3171.71,3641.11) |  |
| Model 1 | 0.53 (0.17,1.62) | 0.3 | 0.58 (0.15,2.23) | 0.4 | 0.31 (0.06,1.68) | 0.2 |
| Model 2 | 0.55 (0.18,1.70) | 0.3 | 0.59 (0.15,2.29) | 0.4 | 0.32 (0.06,1.77) | 0.2 |
| Mg | 18.88 (17.20,20.82) |  | 18.96 (17.17,21.06) |  | 18.69 (17.15,20.43) |  |
| Model 1 | 0.83 (0.33,2.10) | 0.7 | 0.90 (0.28,2.89) | 0.9 | 0.83 (0.22,3.12) | 0.8 |
| Model 2 | 0.81 (0.32,2.05) | 0.7 | 0.88 (0.27,2.85) | 0.8 | 0.79 (0.21,3.01) | 0.7 |
| K | 144.11 (131.14,161.84) |  | 138.79 (124.76,157.75) |  | 147.77 (135.14,16) |  |
| Model 1 | 0.92 (0.38,2.20) | 0.8 | 1.12 (0.40,3.16) | 0.8 | 0.76 (0.20,2.96) | 0.7 |
| Model 2 | 0.97 (0.40,2.33) | 0.9 | 1.14 (0.40,3.25) | 0.8 | 0.77 (0.20,2.98) | 0.7 |
| Ca | 83.00 (74.83,90.19) |  | 81.75 (73.83,90.16) |  | 83.21 (75.17,89.56) |  |
| Model 1 | 0.55 (0.23,1.32) | 0.2 | 0.39 (0.13,1.19) | 0.1 | 0.80 (0.23,2.85) | 0.7 |
| Model 2 | 0.56 (0.23,1.35) | 0.2 | 0.39 (0.13,1.19) | 0.1 | 0.79 (0.22,2.82) | 0.7 |
| Mn | 0.73 (0.73,2.14) |  | 0.73 (0.73,1.97) |  | 0.73 (0.73,2.17) |  |
| Model 1 | 0.86 (0.70,1.06) | 0.2 | 1.00 (0.77,1.31) | 1 | 0.82 (0.61,1.11) | 0.2 |
| Detectable (Exposure/Non-exposure) | 104/422 | 0.2 | 62/216 | 1 | 56/231 | 0.3 |
| Undetectable (Exposure/Non-exposure) | 152/532 |  | 112/384 |  | 71/247 |  |
| Model 2 | 0.87 (0.70,1.07) | 0.2 | 1.00 (0.77,1.31) | 1 | 0.82 (0.61,1.10) | 0.2 |
| Detectable (Exposure/Non-exposure) | 104/422 | 0.2 | 62/216 | 1 | 56/231 | 0.3 |
| Undetectable (Exposure/Non-exposure) | 152/532 |  | 112/384 |  | 71/247 |  |
| Fe | 948.88 (713.21,1230.80) |  | 996.85 (775.96,1273.95) |  | 891.40 (680.17,1195.93) |  |
| Model 1 | 1.15 (0.79,1.67) | 0.5 | 0.94 (0.60,1.49) | 0.8 | 1.56 (0.93,2.62) | 0.1 |
| Model 2 | 1.14 (0.79,1.66) | 0.5 | 0.95 (0.60,1.52) | 0.8 | 1.55 (0.92,2.62) | 0.1 |
| Co | 0.23 (0.14,0.38) |  | 0.19 (0.11,0.29) |  | 0.30 (0.19,0.46) |  |
| Model 1 | 0.86 (0.71,1.05) | 0.1 | 0.81 (0.63,1.03) | 0.1 | 0.90 (0.65,1.24) | 0.5 |
| Model 2 | 0.88 (0.72,1.07) | 0.2 | 0.82 (0.64,1.05) | 0.1 | 0.91 (0.65,1.26) | 0.6 |
| Cu | 1554.86 (1283.05,1809.89) |  | 1391.69 (1157.43,1666.05) |  | 1685.02 (1486.72,1946.10) |  |
| Model 1 | 1.08 (0.62,1.88) | 0.8 | 0.97 (0.49,1.92) | 0.9 | 1.73 (0.67,4.47) | 0.3 |
| Model 2 | 1.05 (0.60,1.83) | 0.9 | 0.94 (0.47,1.86) | 0.8 | 1.71 (0.66,4.47) | 0.3 |
| Zn | **580.83 (476.39,703.04)** |  | **634.01 (537.23,757.50)** |  | 525.85 (444.85,630.32) |  |
| Model 1 | **0.47 (0.28,0.81)** | **6.5×10^-3*^** | **0.28 (0.14,0.58)** | **6.0×10^-4*^** | 0.92 (0.41,2.07) | 0.8 |
| Model 2 | **0.47 (0.28,0.81)** | **6.6×10^-3*^** | **0.28 (0.13,0.58)** | **6.0×10^-4*^** | 0.89 (0.40,2.02) | 0.8 |
| As | **1.06 (0.67,1.97)** |  | **0.99 (0.64,1.85)** |  | 1.10 (0.70,2.16) |  |
| Model 1 | **1.32 (1.10,1.58)** | **2.4×10^-3*^** | **1.46 (1.18,1.80)** | **5.0×10^-4^*** | 1.29 (0.99,1.68) | 0.1 |
| Model 2 | **1.35 (1.13,1.62)** | **1.2×10^-3*^** | **1.49 (1.20,1.84)** | **3.0×10^-4^*** | 1.31 (1.00,1.71) | **4.7×10^-2*^** |
| Rb | 346.57 (286.73,409.49) |  | 334.04 (272.73,399.95) |  | 351.02 (291.42,414.72) |  |
| Model 1 | 1.39 (0.79,2.46) | 0.3 | 1.46 (0.73,2.95) | 0.3 | 1.43 (0.63,3.25) | 0.4 |
| Model 2 | 1.48 (0.83,2.64) | 0.2 | 1.57 (0.77,3.18) | 0.2 | 1.46 (0.64,3.32) | 0.4 |
| Sr | **42.29 (34.59,51.30)** |  | **39.20 (32.44,47.01)** |  | 44.66 (36.70,53.83) |  |
| Model 1 | **0.51 (0.31,0.85)** | **9.7×10^-3*^** | **0.38 (0.20,0.73)** | **3.7×10^-3*^** | 0.55 (0.26,1.15) | 0.1 |
| Model 2 | **0.52 (0.31,0.86)** | **1.1×10^-2*^** | **0.39 (0.20,0.74)** | **4.3×10^-3*^** | 0.53 (0.25,1.13) | 0.1 |
| Mo | 2.33 (1.72,3.23) |  | 2.17 (1.60,2.96) |  | 2.57 (1.82,3.42) |  |
| Model 1 | 1.23 (0.88,1.71) | 0.2 | 0.92 (0.61,1.38) | 0.7 | 1.98 (1.20,3.24) | 7.0×10^-3*^ |
| Model 2 | 1.24 (0.88,1.73) | 0.2 | 0.91 (0.61,1.37) | 0.7 | 2.02 (1.23,3.34) | 5.7×10^-3*^ |
| Cd | 0.04 (0.04,0.04) |  | 0.04 (0.04,0.04) |  | 0.04 (0.04,0.04) |  |
| Model 1 | 0.91 (0.67,1.22) | 0.5 | 0.84 (0.58,1.21) | 0.3 | 1.01 (0.66,1.57) | 1 |
| Detectable (Exposure/Non-exposure) | 42/187 | 0.5 | 22/110 | 0.2 | 25/92 | 0.8 |
| Undetectable (Exposure/Non-exposure) | 215/765 |  | 151/490 |  | 103/383 |  |
| Model 2 | 0.91 (0.67,1.22) | 0.5 | 0.83 (0.57,1.21) | 0.3 | 1.00 (0.65,1.56) | 1 |
| Detectable (Exposure/Non-exposure) | 42/187 | 0.4 | 22/110 | 0.2 | 25/92 | 0.9 |
| Undetectable (Exposure/Non-exposure) | 215/765 |  | 151/490 |  | 103/383 |  |
| Sb | 3.39 (0.82,5.90) |  | 3.07 (0.38,5.02) |  | 4.12 (0.47,7.06) |  |
| Model 1 | 0.87 (0.77,0.98) | 2.2×10^-2*^ | 0.90 (0.77,1.05) | 0.2 | 0.89 (0.76,1.05) | 0.2 |
| Model 2 | 0.87 (0.77,0.98) | 2.0×10^-2*^ | 0.90 (0.77,1.05) | 0.2 | 0.89 (0.75,1.05) | 0.2 |
| Ba | **67.61 (1.33,98.94)** |  | 67.54 (1.33,96.82) |  | **75.81 (1.33,102.83)** |  |
| Model 1 | **1.17 (1.08,1.27)** | **1.0×10^-4^*** | 1.09 (0.99,1.19) | 0.1 | **1.23 (1.09,1.40)** | **1.1×10^-3*^** |
| Model 2 | **1.17 (1.08,1.27)** | **1****.0×10^-4^*** | 1.08 (0.99,1.19) | 0.1 | **1.25 (1.10,1.41)** | **7.0×10^-4^*** |
| Hg | 0.60 (0.44,0.87) |  | 0.61 (0.44,0.89) |  | 0.60 (0.44,0.86) |  |
| Model 1 | 0.93 (0.73,1.18) | 0.5 | 0.84 (0.63,1.12) | 0.2 | 1.03 (0.73,1.47) | 0.9 |
| Model 2 | 0.93 (0.73,1.18) | 0.5 | 0.83 (0.62,1.10) | 0.2 | 1.04 (0.73,1.49) | 0.8 |
| Tl | 0.03 (0.02,0.04) |  | 0.02 (0.02,0.03) |  | 0.03 (0.02,0.04) |  |
| Model 1 | 0.95 (0.73,1.24) | 0.7 | 1.23 (0.85,1.77) | 0.3 | 0.83 (0.57,1.20) | 0.3 |
| Model 2 | 0.95 (0.73,1.24) | 0.7 | 1.25 (0.87,1.81) | 0.2 | 0.81 (0.56,1.18) | 0.3 |
| U | 0.01 (0.01,0.04) |  | 0.01 (0.01,0.03) |  | 0.01 (0.01,0.03) |  |
| Model 1 | 0.79 (0.65,0.96) | 1.6×10^-2*^ | 0.76 (0.60,0.96) | 2.3×10^-2*^ | 0.80 (0.59,1.08) | 0.1 |
| Detectable (Exposure/Non-exposure) | 78/380 | 0.1 | 40/212 | 4.5×10^-2*^ | 40/180 | 0.4 |
| Undetectable (Exposure/Non-exposure) | 176/569 |  | 134/385 |  | 86/296 |  |
| Model 2 | 0.80 (0.66,0.96) | 2.0×10^-2*^ | 0.77 (0.60,0.97) | 2.6×10^-2*^ | 0.80 (0.59,1.09) | 0.2 |
| Detectable (Exposure/Non-exposure) | 78/380 | 0.1 | 40/212 | 0.1 | 40/180 | 0.5 |
| Undetectable (Exposure/Non-exposure) | 176/569 |  | 134/385 |  | 86/296 |  |

Logistic regression incorporating binary outcome (preterm birth vs. term birth) was used.

^a^ Serum metal concentration was presented as raw data.

^b^ Model 1: logistic model with adjustment for BMI, parents’ education level, income levels, and marriage age.

^c^ Model 2: additional covariates adjusted including maternal baseline age, secondhand smoking status and number of previous pregnancies.

^*^ False discovery rate (FDR) adjusted q value ≤ 0.05.

**Appendix Figure 6. Generic paradigm for mediation analysis. U, confounder; Marriage age, exposure; Metal exposure, mediator; Preterm birth, outcome.**

**Appendix Table 3. The effects of marriage age on individual metal concentrations for the first trimester and second trimesters.**

| **Trimester** | **Metal** | **marriage age** | | **Child marriage** | |  |
| --- | --- | --- | --- | --- | --- | --- |
|  |  |  |  | **Yes** | **No** |  |
|  |  | Correlation coefficient | *P^a^* | Median (Q_1_, Q_3_) | Median (Q_1_, Q_3_) | *P^b^* |
| 1^st^ | Na | 0.00(-0.01, 0.01) | 0.98 | 3273.04(3045.04,3616.62) | 3340.43(3077.71,3661.29) | 0.23 |
|  | Mg | 0.00(-0.01, 0.00) | 0.37 | 18.96(17.28,21.20) | 19.00(17.05,20.84) | 0.67 |
|  | K | 0.00(0.00, 0.01) | 0.51 | 136.26(124.32,156.46) | 140.23(125.40,158.79) | 0.14 |
|  | Ca | 0.00(-0.01, 0.01) | 0.88 | 80.38(73.08,89.18) | 82.83(74.40,90.99) | 0.23 |
|  | Mn | 0.01(-0.02, 0.03) | 0.64 | 0.73(0.73,1.99) | 0.73(0.73,1.95) | 0.89 |
|  | Fe | 0.01(-0.01, 0.02) | 0.45 | 996.99(782.83,1307.82) | 996.85(769.75,1257.85) | 0.42 |
|  | Co | 0.08(0.05, 0.10) | 3.92×10^-09^ | 0.15(0.09,0.25) | 0.21(0.13,0.32) | 1.97×10^-08^ |
|  | Cu | -0.01(-0.02, 0.00) | 0.19 | 1395.31(1182.66,1659.18) | 1390.41(1137.04,1671.86) | 0.92 |
|  | Zn | 0.01(0.00, 0.02) | 3.95×10^-03^ | 623.92(521.32,734.72) | 643.34(548.99,785.00) | 0.03 |
|  | As | -0.06(-0.09, -0.03) | 1.36×10^-04^ | 1.29(0.76,2.66) | 0.85(0.58,1.44) | 2.08×10^-10^ |
|  | Rb | 0.00(-0.01, 0.01) | 0.47 | 338.78(271.47,403.62) | 331.37(273.50,392.76) | 0.24 |
|  | Sr | 0.02(0.01, 0.03) | 1.56×10^-03^ | 37.24(31.33,43.88) | 41.07(33.38,49.14) | 6.56×10^-07^ |
|  | Mo | -0.01(-0.03, 0.01) | 0.32 | 2.15(1.64,3.04) | 2.18(1.58,2.95) | 0.64 |
|  | Cd | 0.02(0.00, 0.04) | 0.06 | 0.04(0.04,0.04) | 0.04(0.04,0.04) | 0.14 |
|  | Sb | 0.01(-0.03, 0.05) | 0.60 | 3.39(0.89,5.31) | 2.78(0.38,4.57) | 0.17 |
|  | Ba | -0.09(-0.16, -0.02) | 0.02 | 69.00(1.33,94.19) | 64.59(1.33,99.31) | 0.69 |
|  | Hg | 0.02(0.00, 0.05) | 0.10 | 0.57(0.42,0.83) | 0.67(0.46,1.01) | 0.02 |
|  | Tl | -0.01(-0.02, 0.01) | 0.54 | 0.02(0.02,0.03) | 0.02(0.02,0.03) | 0.07 |
|  | U | 0.07(0.04, 0.10) | 6.16×10^-06^ | 0.01(0.01,0.01) | 0.01(0.01,0.05) | 8.39×10^-11^ |
| 2^nd^ | Na | 0.00(-0.01, 0.00) | 0.11 | 3456.88(3241.41,3730.51) | 3354.30(3130.73,3578.10) | 8.93×10^-05^ |
|  | Mg | -0.01(-0.01, 0.00) | 0.04 | 19.15(17.60,20.69) | 18.46(16.71,20.16) | 4.85×10^-04^ |
|  | K | -0.01(-0.02, 0.00) | 0.01 | 152.68(140.44,165.83) | 144.11(132.92,158.88) | 4.45×10^-05^ |
|  | Ca | -0.01(-0.02, 0.00) | 8.77×10^-04^ | 85.66(78.50,92.03) | 81.68(73.1,87.96) | 3.36×10^-07^ |
|  | Mn | 0.00(-0.03, 0.03) | 0.88 | 0.73(0.73,2.26) | 0.73(0.73,2.14) | 0.53 |
|  | Fe | -0.01(-0.03, 0.01) | 0.28 | 971.35(757.15,1232.58) | 838.22(642.48,1174.03) | 8.11×10^-03^ |
|  | Co | 0.05(0.02, 0.08) | 1.64×10^-04^ | 0.26(0.17,0.41) | 0.33(0.21,0.50) | 4.49×10^-04^ |
|  | Cu | -0.01(-0.02, 0.00) | 0.09 | 1671.35(1513.50,1965.40) | 1689.63(1457.85,1938.14) | 0.10 |
|  | Zn | 0.00(-0.01, 0.01) | 0.78 | 519.45(444.25,605.30) | 538.12(445.57,639.36) | 0.33 |
|  | As | -0.09(-0.12, -0.05) | 4.49×10^-07^ | 1.53(0.86,3.15) | 0.94(0.60,1.59) | 2.19×10^-11^ |
|  | Rb | -0.01(-0.02, 0.00) | 0.04 | 359.45(310.80,428.18) | 342.50(285.67,405.76) | 2.62×10^-03^ |
|  | Sr | 0.00(-0.02, 0.01) | 0.49 | 44.11(37.25,52.80) | 45.16(36.06,54.41) | 0.99 |
|  | Mo | -0.01(-0.02, 0.01) | 0.52 | 2.81(2.09,3.58) | 2.31(1.72,3.25) | 1.20×10^-04^ |
|  | Cd | 0.02(0.00, 0.04) | 0.02 | 0.04(0.04,0.04) | 0.04(0.04,0.04) | 1.03×10^-03^ |
|  | Sb | 0.01(-0.05, 0.06) | 0.85 | 4.69(0.90,7.83) | 3.62(0.38,6.42) | 0.06 |
|  | Ba | -0.11(-0.18, -0.03) | 0.01 | 87.26(31.75,109.51) | 61.65(1.33,96.65) | 4.87×10^-07^ |
|  | Hg | 0.01(-0.02, 0.03) | 0.49 | 0.54(0.42,0.79) | 0.63(0.45,0.88) | 0.20 |
|  | Tl | 0.00(-0.03, 0.02) | 0.69 | 0.03(0.02,0.04) | 0.03(0.02,0.04) | 0.94 |
|  | U | 0.03(0.00, 0.07) | 0.04 | 0.01(0.01,0.02) | 0.01(0.01,0.04) | 4.33×10^-04^ |

^a^ Linear regression analysis was performed to evaluate the effects of marriage age on individual metal concentrations for the first trimester and second trimesters.

^b^ Average change of metal concentrations for first and second trimesters between child marriage and no history of child marriage and the corresponding P value for the *t*-test.

**Appendix Table 4.** **Indirect effects of marriage age on risk of preterm mediated through metal elements.**

| Metals | 1^st^ trimester | | | 2^nd^ trimester | | |
| --- | --- | --- | --- | --- | --- | --- |
|  | Indirect effect (95%CI) | *P* | Mediated% | Indirect effect (95%CI) | *P* | Mediated% |
| Na | 1.00(0.98,1.02) | 0.99 | -0.01% | 1.02(0.99,1.04) | 0.57 | -1.01% |
| Mg | 1.00(0.99,1.02) | 0.89 | -0.14% | 1.01(0.98,1.04) | 0.46 | -0.59% |
| K | 1.00(0.99,1.02) | 0.94 | -0.07% | 1.01(0.98,1.05) | 0.61 | -0.83% |
| Ca | 1.00(0.98,1.03) | 0.98 | -0.14% | 1.02(0.97,1.06) | 0.31 | -0.94% |
| Mn | 1.00(0.99,1.01) | 0.95 | -0.01% | 1.01(0.99,1.03) | 0.79 | -0.58% |
| Fe | 1.00(0.98,1.01) | 0.94 | 0.11% | 0.97(0.94,1.01) | 0.19 | 1.67% |
| Co | 0.93(0.85,1.00) | 0.11 | 4.65% | 0.97(0.91,1.03) | 0.60 | 1.81% |
| Cu | 1.00(0.98,1.02) | 0.89 | -0.17% | 0.98(0.96,1.01) | 0.44 | 1.07% |
| Zn | 0.80(0.70,0.92) | 6.3×10^-3^ | 5.67% | 1.00(0.99,1.01) | 0.98 | 0.01% |
| As | 0.84(0.74,0.95) | 2.1×10^-3^ | 6.67% | 0.88(0.80,0.96) | 2.0×10^-3^ | 7.86% |
| Rb | 1.01(0.99,1.03) | 0.62 | -0.47% | 0.98(0.94,1.01) | 0.21 | 1.36% |
| Sr | 0.87(0.78,0.97) | 1.5×10^-2^ | 4.43% | 1.00(0.97,1.02) | 0.91 | 0.04% |
| Mo | 1.00(0.98,1.02) | 0.79 | -0.23% | 0.99(0.94,1.04) | 0.75 | 0.70% |
| Cd | 0.98(0.95,1.02) | 0.37 | 1.13% | 1.00(0.97,1.03) | 0.89 | 0.01% |
| Sb | 0.99(0.97,1.02) | 0.59 | 0.38% | 0.99(0.96,1.01) | 0.53 | 0.79% |
| Ba | 0.96(0.92,1.00) | 0.10 | 2.31% | 0.78(0.66,0.91) | 1.8×10^-3^ | 9.52% |
| Hg | 0.98(0.95,1.01) | 0.22 | 1.36% | 1.00(0.99,1.02) | 0.60 | -0.16% |
| Tl | 0.99(0.97,1.01) | 0.64 | 0.43% | 1.01(0.99,1.03) | 0.83 | -0.64% |
| U | 0.91(0.84,0.98) | 0.02 | 5.89% | 0.97(0.94,1.00) | 0.38 | 1.88% |

OR and 95% CI represent the indirect effects of marriage age on risk of preterm mediated through metal elements using Bootstrap simulation; Mediated% represents the effects of marriage age on preterm birth were mediated by maternal serum.

**Appendix Table 5.** **Odds ratio, PAR% and NNT of child marriage and metal exposure on risk of preterm birth.**

| Trimesters | Variables | OR (95%CI) | *P* | PAR% (95%CI)/per 1 SD unit | NNT(95%CI))/per 1 SD unit |
| --- | --- | --- | --- | --- | --- |
|  | **Child marriage** | **4.18(2.78,6.29)** | **5.71×10^-12^** | **54.23% (42.91%, 65.55%)** | **-4 (-7, -3)** |
| 1^st^ | Na | 0.59(0.13,2.59) | 0.4804 | -7.80% (-31.98%, 16.37%) |  |
|  | Mg | 0.96(0.27,3.41) | 0.9507 | -12.53% (-38.20%, 13.15%) |  |
|  | K | 1.20(0.37,3.92) | 0.7653 | -4.01% (-26.66%, 18.63%) |  |
|  | Ca | 0.38(0.11,1.29) | 0.1208 | -16.65% (-42.49%, 9.20%) |  |
|  | Mn | 1.00(0.75,1.35) | 0.9751 | 0.21% (-18.50%, 18.92%) |  |
|  | Fe | 0.90(0.55,1.47) | 0.6767 | -4.25% (-28.34%, 19.85%) |  |
|  | Co | 0.81(0.62,1.06) | 0.1194 | -5.25% (-27.24%, 16.74%) |  |
|  | Cu | 1.00(0.48,2.08) | 0.9945 | -10.22% (-35.83%, 15.39%) |  |
|  | **Zn** | **0.26(0.12,0.58)** | **9.00×10^-04^** | **-30.24% (-59.35%, -1.14%)** | **11 (9, 20)** |
|  | **As** | **1.53(1.22,1.92)** | **2.00×10^-04^** | **21.02% (2.14%, 39.90%)** | **-18 (-41, -11)** |
|  | Rb | 1.72(0.81,3.65) | 0.1547 | 6.61% (-14.48%, 27.70%) |  |
|  | **Sr** | **0.37(0.19,0.75)** | **0.0053** | **-19.19% (-45.11%, -6.73%)** | **13 (10, 34)** |
|  | Mo | 0.91(0.60,1.40) | 0.6730 | -1.35% (-23.58%, 20.88%) |  |
|  | Cd | 0.82(0.56,1.21) | 0.3234 | -4.87% (-21.14%, 11.40%) |  |
|  | Sb | 0.91(0.76,1.08) | 0.2857 | -15.32% (-42.25%, 11.62%) |  |
|  | Ba | 1.08(0.98,1.20) | 0.1138 | 6.90% (-13.95%, 27.75%) |  |
|  | Hg | 0.81(0.60,1.09) | 0.1616 | -20.03% (-46.03%, 5.98%) |  |
|  | Tl | 1.38(0.92,2.07) | 0.1148 | -2.06% (-25.51%, 21.40%) |  |
|  | U | 0.78(0.60,1.01) | 0.0561 | -11.60% (-31.57%, 8.36%) |  |
| 2^nd^ | Na | 0.33(0.06,1.82) | 0.2055 | -5.26% (-32.41%, 21.89%) |  |
|  | Mg | 0.83(0.22,3.21) | 0.7903 | -3.76% (-30.41%, 22.88%) |  |
|  | K | 0.81(0.20,3.21) | 0.7600 | -13.52% (-44.39%, 17.35%) |  |
|  | Ca | 0.97(0.26,3.63) | 0.9599 | -3.42% (-30.27%, 23.43%) |  |
|  | Mn | 0.84(0.62,1.14) | 0.2588 | -10.44% (-37.42%, 16.54%) |  |
|  | Fe | 1.55(0.92,2.61) | 0.1020 | 1.96% (-24.82%, 28.73%) |  |
|  | Co | 0.91(0.65,1.26) | 0.5684 | -11.05% (-37.44%, 15.33%) |  |
|  | Cu | 1.59(0.61,4.16) | 0.3479 | 0.94% (-23.93%, 25.81%) |  |
|  | Zn | 0.85(0.36,2.02) | 0.7166 | -5.99% (-32.35%, 20.37%) |  |
|  | As | 1.35(1.03,1.76) | 0.0294 | 14.30% (-9.25%, 37.85%) |  |
|  | Rb | 1.55(0.67,3.57) | 0.3081 | 0.15% (-25.68%, 25.98%) |  |
|  | Sr | 0.57(0.27,1.22) | 0.1453 | -0.07% (-24.80%, 24.66%) |  |
|  | Mo | 2.07(1.25,3.42) | 0.0048 | 22.82% (2.83%, 42.81%) |  |
|  | Cd | 1.00(0.64,1.56) | 0.9969 | 2.38% (-14.77%, 19.53%) |  |
|  | Sb | 0.89(0.75,1.05) | 0.1747 | -4.73% (-31.47%, 22.00%) |  |
|  | **Ba** | **1.25(1.10,1.43)** | **8.00×10^-04^** | **27.31% (7.95%, 46.68%)** | **-37 (-90, -22)** |
|  | Hg | 1.02(0.71,1.47) | 0.8970 | -2.60% (-27.17%, 21.98%) |  |
|  | Tl | 0.82(0.56,1.19) | 0.2899 | 1.82% (-23.02%, 26.67%) |  |
|  | U | 0.77(0.56,1.06) | 0.1105 | -3.24% (-25.27%, 18.79%) |  |

OR and 95% CI represent the effects of marriage age and metal exposure on risk of preterm using logistic regression; PAR% represents the population attribute risk percentage.

The NNT is defined as the number of patients who would need to be treated on average to prevent 1 additional bad outcome or achieve 1 desirable outcome in a given period of time.
